# Techno-functionality of pea protein isolate: Influence of ultrasound and high-pressure homogenization modification methods

**DOI:** 10.1016/j.ultsonch.2025.107321

**Published:** 2025-03-23

**Authors:** Fereshte Bahmanyar, Mitra Pashaei, Kooshan Nayebzadeh, Ali Dini, Leila Mirmoghtadaie, Hedayat Hosseini

**Affiliations:** a Department of Food Science and Technology, National Nutrition and Food Technology Research Institute, Faculty of Nutrition Sciences and Food Technology, Shahid Beheshti University of Medical Sciences, Tehran, Iran; b Pistachio Safety Research Center, Rafsanjan University of Medical Sciences, Rafsanjan, Iran; c Vali-e-asr University of Rafsanjan, Rafsanjan, Iran

**Keywords:** Pea protein isolate, Ultrasound, High pressure homogenization, Functional properties, Structural properties

## Abstract

Pea protein isolate (PPI) was modified using high-pressure homogenization (HPH) at three pressure levels (60, 80, and 100 MPa) for three cycles and ultrasound (US) at three power levels (100, 200, and 300 W) for 10 min. Results showed that both techniques significantly increased solubility, oil holding capacity, emulsifying activity and foam capacity. Ultimately, a homogenization pressure of 100 MPa (HPH 100) and an ultrasound power of 300 W (US 300) were identified as the optimal treatments. HPH and US treatments led to a significant reduction in particle size and an increase in the surface charge of the samples. FTIR spectroscopy revealed changes in hydrogen bonding and the secondary structure of PPI after HPH and US treatment. Also, SEM imaging showed that the spherical shape of PPI transformed into heterogeneous sheet structures. Additionally, the comparison of HPH and US techniques revealed that the HPH resulted in a greater reduction in size and a greater increase in solubility and FC compared to the US. On the other hand, the US technique showed greater EAI and thermal stability. Therefore, both HPH and US are effective in altering the PPI structure to enhance its functional properties.

## 1 Introduction

In recent years, plant proteins have become a promising food source due to their low cost, easy accessibility, and environmental sustainability for the growing global population [21]. Among plant proteins, pea protein has attracted significant interest due to its low allergenicity, high nutritional value, low cost, wide accessibility, and non-GMO status [18,29]. In addition, the world production of pea protein is about 10 million tons per year [28]that contains 18 %–25 % of water-soluble protein (albumin) and 55 %–65 % of salt-soluble protein (globulin). It is a rich source of tryptophan, threonine, and lysine making it a promising protein compared to other plant proteins like soybean [24,26]. Pea protein is widely used in the food industry across various fields, including meat analogs, dairy products and their alternatives, bakery applications, beverages, sauces, and as an encapsulating wall material [24](. Nevertheless, the use of pea protein in the food industry is limited due to its weak functional properties, such as solubility, gelation, and emulsification [18,29]. The functional properties of pea protein can be improved during application of food processing techniques [11].

High-pressure homogenization (HPH), a mild physical process, is used to increase the stability of the emulsion inactivate enzymes and bacteria, and extract beneficial compounds. Nowadays, HPH has attracted significant attention for its ability to alter the protein structure and modifying its functional properties [18]. During the HPH process, the fluid is passed through a narrow gap and when it reaches to atmospheric pressure, a pressure drop occurs, which leads to shear forces, turbulence, cavitation phenomenon, and increase of fluid temperature [2,27]. HPH treatment can disrupt protein aggregates, alter the tertiary and quaternary structures of protein and modify its conformation, which leads to positive effects on emulsifying activity, foaming capacity, gelling and solubility properties [18,31]. Ultrasound treatment (US) is another common physical technology that can be used to modify the functional properties of protein [13]. It is known as a non-toxic, safe and eco-friendly technology [22]. Ultrasonic treatment with low frequency and high intensity (20 KHz), through the cavitation leads to disruption and size reduction of protein aggregates resulting in interactions and changes in protein structure [9,13]. Therefore, changes in protein structure using HPH and US processes lead to more exposure of reactive groups such as hydrophobic and sulfhydryl groups, on the protein molecule surface, that can be associated with improvements in protein functional properties, such as solubility, emulsifying and foaming capacity [29]. In this context, many studies have reported that HPH and US treatments have a good potential to modify the functional properties of faba bean protein [30], lentil protein [23], insoluble pea protein [21], sunflower protein [16]and soy protein isolate [8]. According to literature, the functionality of protein can be improved by increasing the intensity of the process. However, excessive process intensity can lead to protein aggregation and a reduction of functionality [18]. Since the effects of physical techniques depend on the intensity of the process, it seems necessary to optimize these techniques. Therefore, in this study, after selecting samples with better functional properties, two techniques were compared, making this study exciting.

The aim of this study is to optimize two techniques of HPH and US in order to improve the functional and structural properties of pea protein isolate. The optimum sample from each technique was selected based on solubility, water and oil holding capacity, emulsifying and foaming properties. Additional tests were performed to evaluate the properties of the optimum pea protein isolate and compare them with those of the untreated sample.

## 2 Materials and methods

### 2.1 Materials

Pea protein isolate (PPI) (80 % protein, 5.5 % fat, 2.6 % carbohydrates, 4.1 % fiber, and 1.9 % salt) was obtained from the MYVEGAN brand (MYVEGAN Pea Protein Isolate Powder, made in the United Kingdom). Canola and sunflower oil (Ladan, Iran) were purchased from a local supermarket. Chemical materials such as SDS was obtained from Merck company (Germany).

### 2.2 High pressure homogenization (HPH) and ultrasound (US) treatments of PPI

The PPI (5 % w/v) was dispersed in distilled water and mixed on a magnetic stirrer at 25 ^◦^C for 1 h. The PPI suspensions were treated with HPH (Panda Plus 1500; GEA Niro Soavi, Parma, Italy) for three cycles at 60, 80 and 100 MPa (samples including: HPH 60, HPH80, HPH 100 respectively). The beaker containing the sample was immersed in an ice-water bath to prevent the temperature from increasing during HPH treatments [23].

The PPI dispersions (5.0 %, w/v) were prepared by dissolving PPI powder in distilled water and stirring for 1 h at 25 ^◦^C. Then 100 mL of the PPI dispersion was treated with ultrasonic probe (UP-400-A, Iran) at 20 kHz under three power levels of 100, 200, 300 W for 10 min (working for 3 s, and stopping for 2 s, samples including: US100, US 200, US 300 respectively). The beaker containing 100 mL PPI dispersions was placed in an ice-water bath to control the temperature below 35 ^◦^C during the ultrasonic treatment [13].

HPH and US samples were prepared in triplicate, freeze-dried, and stored in a refrigerator until further analysis. They were then compared with the untreated sample (Native PPI, N).

### 2.3 Solubility

The solubility of the samples was determined according to [18] with some modifications. The PPI powders were dispersed in deionized water (1 %w/v) and stirred for 1 h and at room temperature. The dispersions were centrifuged at 9000 rpm for 30 min. The supernatant was removed, and the precipitate was dried in a vacuum oven. The solubility was calculated by Eq (1):

(1) Solubility(%)=(Initialweight-Driedprecipitateweight)/Initialweight×100

### 2.4 Water and oil holding capacity

PPI was mixed with distilled water or sunflower oil (1:10) and vortexed for 1 min. The suspension was kept motionless for 30 min and then centrifuged at 8000 rpm for 20 min. The supernatant was removed and tube was inverted for 5 min to drain protein sediment. Water holding capacity (WHC) and oil holding capacity (OHC) were calculated using the following Eq. (2) [2,14].

(2) WHC/OHC=(MassofoilorwaterbindingtoPPI(g)/MassofinitialPPI(g))

### 2.5 Emulsifying properties

Emulsifying activity index (EAI) of untreated and treated PPI was determined according to [30] with some modifications. Briefly, 10 mL of canola oil was added to 30 mL of PPI dispersion (5 mg/mL) and homogenized using a high-speed homogenizer (IKA T25 Digital Ultra-Turrax, Germany) at 10000 rpm for 2 min. Immediately, 100 μl of emulsion was taken from the bottom of the beaker and added to 10 mL 0.1 % SDS solution and recorded the absorption at 500 nm (PERKIN ELMER, Lambda 2). The SDS solution 0.1 % was introduced as blank. EAI was calculated by the following Eq. (3):

(3) $$\mathrm{EAI}\;\left(m^{2}/g\right)\;=\;\left(2\times 2.303\times A\times D\right)\;/\left(l_{0}^{4}\times c\times \phi \right)$$

Where A is the absorbance at 0 min, D is the dilution factor (100), c is the protein concentration prior to formation of emulsion (g/mL), ϕ is the oil volume fraction of the emulsion system (0.25).

### 2.6 Foaming properties

Foam capacity (FC) and foam stability (FS) of PPI were measured according to [17] with slight modifications. One gram of PPI powder was mixed with 33.3 mL of distilled water and stirred using a magnetic stirrer for 45 min at ambient temperature. Then, the suspension was homogenized with Ultra Turrax (IKA T25-Digital Ultra Turrax, Staufen, Germany) at 10,000 rpm for 90 s, and immediately transferred into a 100 mL measuring cylinder, and the total volume of foam was recorded at 0, 10 and 30 min. Foam volume at 0 min was considered as FC and FS was calculated using the following Eq. (5):

(5) FS(%)=(Foamvolumeateachtime/Volumebeforehomogenizing)×100

### 2.7 Particle size distribution and zeta potential

Particle size distribution and zeta potential (ζ) were measured using dynamic laser light scattering (DLS, Zetasizer Nano ZS, Malvern, UK). The PPI powder was diluted to 5 mg/mL and placed in a cuvette. The protein size parameter is reported the as Z-average.

### 2.8 Differential scanning calorimetry (DSC)

The thermal properties of PPI were determined using DSC (STAR^e^, Mettler Toledo, Switzerland) method. About 8 mg of the protein isolate was heated from 10-180 °C at a rate of 10 °C/min [16].

### 2.9 Fourier-transform infrared spectroscopy (FTIR)

FTIR spectroscopy of PPI was recorded using an FT-IR spectrometer (Agilent, Cary 630 FTIR, US) with 60 scans in the range of 4000 to 650 cm^−1^ and resolution of 8 cm^−1^ [13].

### 2.10 Scanning electron microscopy (SEM)

The morphology of the PPI powder was observed using a scanning electron microscope (MIRA 3 LMU, TESCAN, Czech Republic) at an accelerating voltage of 15 KV. The samples’ surfaces were coated with gold before taking images of the samples (Xiong, [28].

### 2.11 Statistical analysis

All measurements were performed in at least triplicate, and the results are presented as mean values ± standard deviation. SPSS Statistics version 25 software was applied to analyze the data. Statistical evaluations were performed by one-way analysis of variance (ANOVA), and subsequent Duncan’s multiple range test was used to determine the significance (α = 0.05).

## 3 Results and discussion

### 3.1 Solubility

The solubility of PPI treated with HPH and US techniques is indicated in Table 1. Both HPH and US techniques caused a significant increase in protein solubility. The HPH100 and US300 showed the highest solubility, increasing from 14.53 % in untreated PPI to 37.27 % and 18.09 % in HPH100 and US300 samples, respectively. The increase in protein solubility can be attributed to the reduction of protein particle size under the HPH and US processes and increase of the protein-water interactions [19]. Saricaoglu [23] reported that the application of HPH up to 100 MPa led to an increase in the lentil protein isolate solubility through the unfolding of the protein structure and increasing protein interactions. Also, [30] observed the solubility of faba bean proteins increased to 99 % after treatment with HPH at 30 kpsi. Moreover, Malik, Sharma and Saini [16] reported changes in protein conformation and conversion of the insoluble aggregates to soluble protein which led to an increase in sunflower protein solubility after ultrasound treatment. These results are consistent with the results reported by Gao, Rao and Chen [4] who noted that the solubility of commercial PPI was improved from 7.2 to 58.4 mg/mL after a sequential US treatment at power of 150 W. Similar results were recorded for the solubility of soy protein isolate [8], sunflower protein isolate [16], and grass pea protein isolate [19]after US treatment. Also in this context, [6] reported that high-intensity ultrasound, based on inherent protein characteristics, is a useful technique for improving protein solubility and other functional properties.

**Table 1:** Functional properties of untreated and treated PPI.

	**Treatment**	**Solubility** (%)	**WHC** (g/g)	**OHC** (g/g)	**EAI** (m^2^/g)
	N	14.53 ± 0.52^c^	3.21 ± 0.12^a^	1.00 ± 0.09^b^	20.66 ± 0.19^b^
**HPH (MPa)**	HPH 60	31.35 ± 1.88^b^	1.77 ± 0.01^b^	2.30 ± 0.09^a^	20.71 ± 0.16^b^
	HPH 80	37.22 ± 0.26^a^	1.72 ± 0.01^b^	2.40 ± 0.11^a^	20.81 ± 0.13^b^
	HPH100	37.27 ± 1.73^a^	1.73 ± 0.02^b^	2.44 ± 0.07^a^	22.18 ± 0.27^a^
	N	14.53 ± 0.52^b^	3.21 ± 0.12^ab^	1.00 ± 0.09^c^	20.66 ± 0.19^d^
**US** **(W)**	US 100	17.27 ± 0.78^a^	3.05 ± 0.02^b^	1.75 ± 0.02^b^	22.66 ± 0.40^c^
	US 200	17.62 ± 0.45^a^	3.18 ± 0.01^ab^	1.86 ± 0.11^b^	26.24 ± 0.36^b^
	US 300	18.09 ± 2.18^a^	3.33 ± 0.03^a^	2.19 ± 0.04^a^	29.68 ± 0.41^a^

Different letters in the same column indicate significant differences (P < 0.05) between different treatments.

Therefore, the increase in solubility after HPH and US in this study is attributed to the decrease in the particle size of proteins, as confirmed by the results presented in Fig. 2. Moreover, a comparison between HPH and US revealed that the HPH technique had nearly twice the effect on sample solubility compared to ultrasound. The increased solubility of HPH-treated samples may be attributed to their smaller particle size, as also confirmed in Fig. 2.

### 3.2 Water and oil holding capacity

Table 1 shows the results of WHC and OHC of untreated and treated PPI. The HPH and US techniques did not have a positive effect on the WHC of the PPI, but the OHC increased significantly. OHC of protein has an important role in the physical properties of mixed or viscous foods [12]. OHC was increased from 1 g/g in untreated PPI to 2.44 and 2.19 g/g in HPH100 and US300 samples respectively. This increase may be due to the denaturation and unfolding of the protein structure and exposure of hydrophobic groups, which leads to the formation of a network and trapping of oil droplets in the protein structure [14]. Similarly, [2] reported that the HPH treatment could affect OHC of native pea protein and was significantly increased at the pressure of 100 MPa due to exposure of hidden hydrophobic groups on the protein molecule surface. In addition, [22] observed an increase in oil binding capacity and a decrease in water binding capacity of ultrasound treated soy protein isolate. They noted that the denaturation of proteins and the exposure of hydrophobic groups under sonication led to improved physical oil entrapment by protein molecules [22].

### 3.3 Emulsifying properties

Emulsifying properties represent the ability of a protein to adsorb at oil/water interface and depend on certain protein characteristics such as surface hydrophobicity, surface charge, solubility, and molecular flexibility [17,25]. The emulsifying activity index (EAI) is evaluated as one of these emulsifying properties [25]. Table 1 shows the EAI of PPI modified by HPH and US techniques. The EAI of treated PPI increased significantly after HPH and US techniques. The EAI increased from 20.66 (m^2^/g) in untreated PPI to 22.18 (m^2^/g) in HPH100 treatment. In this context, Saricaoglu [23] reported that the EAI of lentil protein isolate was increased during the application of 50 to 100 MPa pressure that can be due to the unfolding of the protein structure and increase in hydrophobic and hydrophilic interactions. The EAI of PPI treated with US was improved (P < 0.05) with increase in ultrasound power up to 300 W and the highest EAI was observed in the sample treated at 300 w for 10 min. Previous studies have noted that, in the ultrasonic process, the cavitation phenomenon causes particle size reduction, protein denaturation, and an increase in hydrophobicity, resulting in enhanced oil–water interfaces and changes in the protein’s emulsifying properties [17]. Therefore, in this study, the increase in EAI in HPH 100 and US300 samples can be due to the decrease in the size of the proteins, changes in protein structure and the increase in hydrophobic and hydrophilic interactions. Additionally, these findings confirm the results of solubility and OHC in the previous step. In this line, [14] reported that the application of HPH treatment led to a significant increase in the EAI of soy protein concentrate compared to the native protein after enzymatic digestion. Similarly, [20] reported that US treatment led to an increase in EAI of millet protein concentrate. Contrary to these results, [30] observed a decrease in the EAI of faba bean protein treated by HPH at 30 kpsi), and they attributed this result to the flocculation effect.

Moreover, the comparison of HPH and US revealed that the effect of US technique on the EAI of the samples was greater than that of the HPH technique.

### 3.4 Foaming properties

Table 2 shows the foaming properties of PPI after using HPH and US techniques. The homogenization that occurs during the HPH and US processes has an important effect on foaming properties of proteins [17]. The FC of PPI was increased (P < 0.05) with the increase in the pressure of HPH and power of US, and the highest FC was recorded for HPH100 and US300 samples. These results are also in agreement with the findings of [17], who observed the FC of wheat gluten and whey protein increased after US treatment at 100 W for 10 min. In addition, the FS of PPI increased significantly after HPH and US process until 30 min. In this context, [21] observed that the FS of insoluble pea protein isolate significantly increased after HPH process at pressures of 60 and 120 MPa due to further unfolding and structure rearrangement of the protein at interface after reaching the air–water interface.

**Table 2:** Foam properties of untreated and treated PPI.

	**Treatment**	**FC** (ml)	**FS 10** (%)	**FS 30** (%)
	N	12.0 ± 0.0^d^	30.04 ± 0.01^c^	21.03 ± 0.0^c^
**HPH (MPa)**	HPH 60	27.01 ± 0.01^c^	54.05 ± 0.0^b^	21.04 ± 0.03^c^
	HPH 80	28.00 ± 0.0^b^	54.06 ± 0.01^b^	27.04 ± 0.02^b^
	HPH100	31.00 ± 0.0^a^	55.55 ± 0.0^a^	30.05 ± 0.03^a^
	N	12.0 ± 0.0^d^	30.04 ± 0.01^d^	21.03 ± 0.0^d^
**US** **(W)**	US 100	12.6 ± 0.14^c^	31.54 ± 0.01^c^	24.04 ± 0.01^c^
	US 200	14.0 ± 0.0^b^	36.65 ± 0.02^b^	33.04 ± 0.01^b^
	US 300	19.0 ± 0.0^a^	45.05 ± 0.01^a^	37.55 ± 0.01^a^

Different letters in the same column indicate significant differences (P < 0.05) between different treatments.

So, according to Table 2, the FC of untreated PPI increased by 2.5 and 1.5 times after the 100 MPa HPH process and 300 W US treatment, respectively, which confirms the results of solubility. It can be related to the rearrangement of the protein structure, changes in particle size and surface charge of the protein. Moreover, the comparison between the HPH and US processes showed that the samples treated with HPH had higher FC than US treated sample. According to the results of section 3.5, the HPH100-treated sample had the smallest size and the highest surface charge, which may lead to improved water/air interactions and increased FC.

**Fig. 1:**
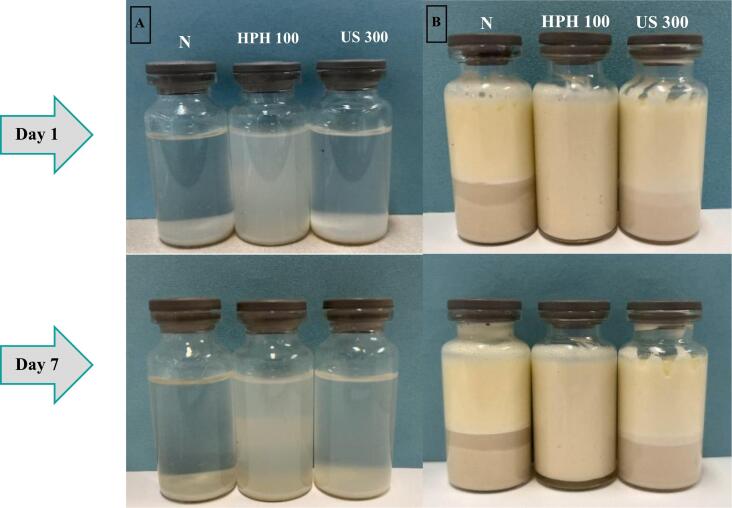
Images of untreated and treated PPI dispersions (A) and emulsions (B) with HPH and US.

Finally, the HPH100 and US300 samples were selected due to better performance in solubility, OHC, emulsifying and foaming properties compared to other samples. Further, the study was carried out on selected samples. Subsequently, water dispersibility of proteins was evaluated. As Fig. 1.a shows, the HPH process improved dispersion properties of proteins. The HPH 100 sample formed a more stable protein dispersion, and this stability was maintained for up to one week after preparation. The stability of the emulsion was also evaluated. As Fig. 1.b shows, the HPH100-treated sample formed a more stable emulsion which remained stable for a week.

### 3.5 Particle size distribution and zeta potential

**Fig. 2:**
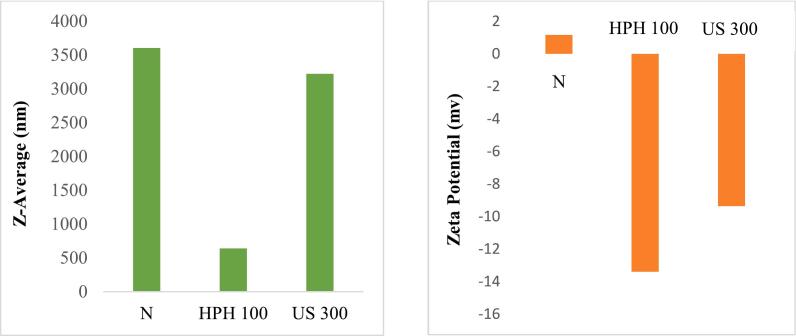
Particles size average and ζ- potential of untreated PPI and treated PPI with HPH and US.

The effects of HPH and US treatments on particle size and ζ-potential of PPI are shown in Fig. 2. The size of the protein particles completely affects the functional properties of the protein [7]. HPH and US treatments reduced the average particle size of PPI from 3606 nm to 640.7 nm and 3224 nm, respectively. In line with these results, many researchers [16], Xiong, [13,19,21,28,29]have reported that HPH and US treatments lead to a decrease in protein particle size. The impact of cavitation and shear forces during HPH and US treatments led to disaggregation of proteins through the disruption of electrostatic interactions, hydrophobic interactions, and hydrogen bonds. This phenomenon resulted in a reduction in protein size and an increase in surface area [19,21,29].

The ζ-potential indicates the surface charge of protein and can express the stability of the protein solution [21]. As can be seen in Fig. 2, the HPH and US treatments created a negative charge on the surface of the protein. Additionally, the HPH process caused a greater increase in the negative charge (−13.4 mV) on the protein surface compared to the US treatment (−9.35 mV). Exposure of non-polar hydrophobic groups due to the breakdown of protein aggregates results in an increase in the negative charge on the protein surface. An increase in negative charge leads to increased electrostatic repulsion and a decrease in the adsorption rate [19]. In this context, [21] reported that a more negative charge on protein surface can lead to electrostatic repulsion and prevent the formation of protein aggregates. Consistent with these results, [13] observed the surface charge of soy protein isolate increased after ultrasonic treatment. This result could be due to the exposure of polar amino acids which leads to inhibition of protein aggregation and, consequently, a decrease in protein particle size. Furthermore, an inverse relationship between particle size and zeta potential was observed, with the treated sample exhibiting a smaller size and a higher surface charge. These findings align with the results obtained in the previous steps.

In comparing the HPH and US samples HPH 100 resulted in the smallest size and the most negative surface charge. These results were in accordance with the increased solubility and FC in HPH 100 sample.

### 3.6 Differential scanning Calorimetry

The DSC technique is used as a quick and easy method to evaluate the thermal stability of proteins indicating the protein’s thermal resistance to denaturation [20]. DSC determines T_peak_, which shows the denaturation temperature of proteins [17].

**Fig. 3:**
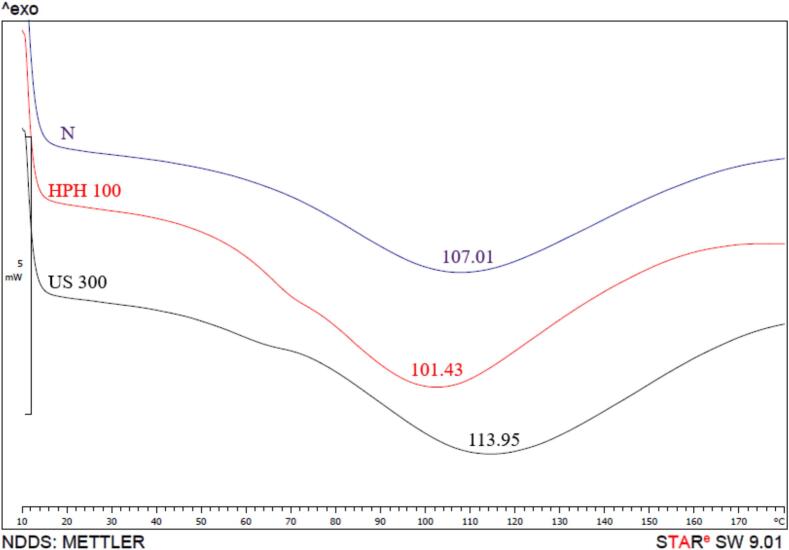
DSC thermograms of untreated PPI and treated PPI with HPH and US.

As shown in Fig. 3, before the main peak in HPH100 and US300 treatments, a slight depression is observed, which is due to the presence of different fractions such as legumin and vicilin in the protein. However, in the untreated sample, only one denaturation peak was seen that may be due to the overlapping of these fractions. The untreated PPI showed an endothermic peak at 107.01 °C that decreased from 107.01 to 101.43 °C (Fig. 3) after HPH processes. Consistent with this result, [2] observed that the T_ONSET_ of pea protein isolate significantly decreased after treatment with HPH. The destruction of bonds and interactions within the protein during high pressure makes it prone to unfolding, and the unfolded protein is less thermally stable than the native one [2]. Moreover, Malik, Sharma and Saini [16] reported that the transition temperature (Td) of sunflower protein isolate decreased after ultra-sonication after 20 min due to changes of protein conformation.

On the other hand, Fig. 3 shows that T_peak_ increased after US treatment, indicating a significant improvement in the thermal stability of US300. According to a study by [15], this increase may be attributed to the enhanced β-sheet structure, which is associated with structural changes in the protein following US treatment.

### 3.7 Fourier-transform infrared spectroscopy

Different compounds have different infrared absorption due to their different molecular structures, so they will record different infrared absorption spectra [13]. Therefore, FTIR can show structural differences among different treatments. The FT-IR spectrum represents the molecular interactions and secondary structure of the protein [1]. The Main and most important bands in the FT-IR spectrum of proteins used to distinguish secondary structures are Amide I and Amide II [3].

**Fig. 4:**
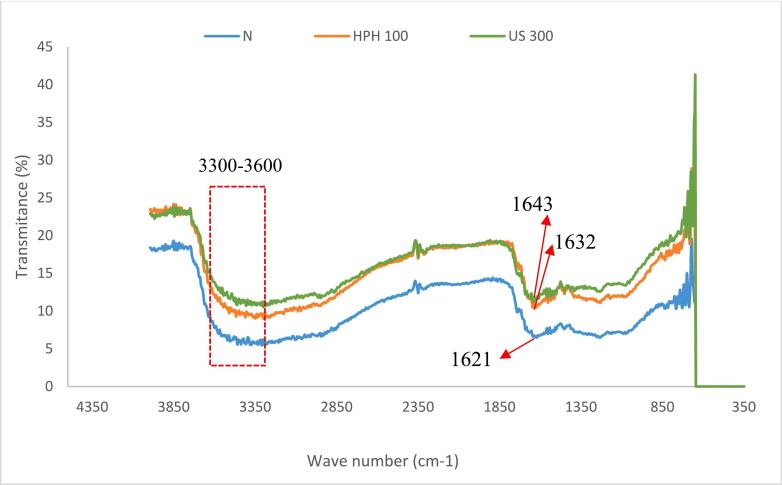
FTIR spectra of untreated PPI and treated PPI with HPH and US.

Amide I band covers the wavelength range of1600-1700 cm^−1^ and is related to the stretching vibrations of the C=O bond, and Amide II band is absorbed at the range of 1480–1575 cm^−1^ and primarily indicates the bending vibrations of the N—H bond [3,10]. Since the C=O and N—H bonds are involved in the formation of hydrogen bonds between different elements of the secondary structure of the protein, the changes in the position of the Amide I and Amide II bands indicates changes in the secondary structure of the protein [3]. As shown in Fig. 4, the peak spectra of Amide I shifted from 1621.39 cm^−1^ to 1632.57 cm^−1^ after the HPH process and to 1643.76 cm^−1^ after US treatment.

Moreover, the peaks in the wavelength range of 3300 to 3600 cm^−1^ are related to N–H and O–H stretching vibrations and were used to assess the intensity of intramolecular and intermolecular hydrogen bonds [5]. According to Fig. 4, the changes in location and/or disappearance of the peaks in the region of 3300 to 3600 cm^−1^ after HPH processing and US treatment are related to alterations of hydrogen bonds. In this line, [30] reported that HPH treatment had a specific impact on the hydrogen bonds, which led to changes in the secondary structure faba bean protein.

Therefore, the FTIR spectroscopy results of the present study showed that the hydrogen bonds and secondary structure of PPI were altered after modification through HPH and US treatment.

### 3.8 Scanning electron microscopy (SEM)

**Fig. 5:**
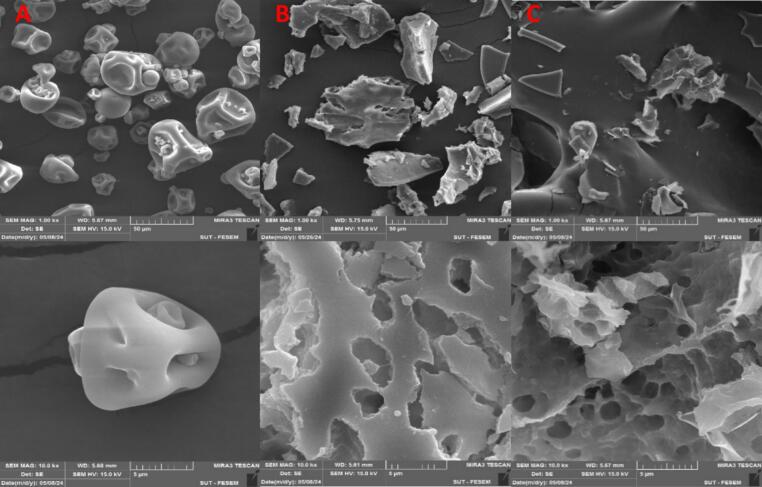
SEM images of untreated PPI (A) and treated PPI with HPH (B) and US (C) at two magnifications of 1.00 (top) and 10.0 (bottom) kx.

The changes in the microstructure of PPI before and after treatment with HPH and US were observed by SEM. Fig. 5 shows the spherical shape of untreated PPI with large holes, was destroyed after modification with HPH and US. The globular structure of untreated PPI was transformed into heterogeneous sheet structures. Additionally, after modification, the PPI particles appeared in various sizes and many particles were smaller than those in the untreated sample; which was in agreement with the results of average particle size in the previous step. Consistent with these results, [32] observed, the spherical structure of the soy protein isolates turned into a clump structure after HPH treatment. They also noted these clumps formed at different sizes and shapes.

Moreover, untreated PPI had a smooth surface, but the treated PPI exhibited a rough surface with sharp edges. Similarly, [21] observed spherical shapes with smooth surface and rough surface for soluble-PPI and insoluble-PPI. These authors also observed two types of morphologies, such as aggregate-like clusters and flake-like fragments in PPI after HPH treatment [21].

## 4 Conclusion

The current study aimed to optimize the HPH and US techniques to modify and improve the properties of native PPI and then evaluate and compare the impact of these two techniques on the structure and functional properties of PPI. HPH and US techniques improved the structural and functional properties of PPI. Based on the results of this study, the best pressure and power for modifying PPI through HPH and US were 100 MPa at three cycles and 300 W for 10 min, respectively. The Z-average showed that these treatments led to a decrease in particle size of the protein. The reduction in the particle size of PPI resulted in an increase in the surface area and an increase in the negative surface charge. Reducing the size and repulsion of the negative charge prevents the coagulation of the protein, so it led to increased solubility, emulsifying activity and foam capacity. Moreover, shear force, turbulence, cavitation phenomenon during HPH and US treatments caused disruption of non-covalent bonds and changes in the secondary structure of the protein. Therefore, the hydrophobic groups buried inside the structure were more exposed, leading to increased surface hydrophobicity and OHC. Also, the FTIR spectroscopy confirmed that the hydrogen bonds and secondary structure of PPI changed after HPH and US treatment. The SEM images showed that the spherical PPI particles were crushed and turned into sheet structures after treatment. Accordingly, the results of this study suggested that the HPH technique resulted in a greater reduction in size and a greater increase in solubility and FC compared to the US technique. On the other hand, the US technique showed greater EAI and thermal stability. Overall, these two techniques improve the structure and functional of PPI and making it more efficient in food science.

## CRediT authorship contribution statement

**Fereshte Bahmanyar:** Writing – original draft, Methodology, Investigation, Formal analysis, Conceptualization. **Mitra Pashaei:** Writing – review & editing. **Kooshan Nayebzadeh:** Methodology, Investigation. **Ali Dini:** Validation, Investigation. **Leila Mirmoghtadaie:** Writing – review & editing, Supervision, Project administration, Methodology, Conceptualization. **Hedayat Hosseini:** Supervision, Project administration, Methodology, Conceptualization.

## Funding

This work is based upon research funded by Iran National Science Foundation (INSF) under project No.4028283.

## Declaration of competing interest

The authors declare that they have no known competing financial interests or personal relationships that could have appeared to influence the work reported in this paper.
